# Effect of statin on progression of symptomatic basilar artery stenosis and subsequent ischemic stroke

**DOI:** 10.1371/journal.pone.0183798

**Published:** 2017-10-11

**Authors:** Kyu Sun Yum, Jun Young Chang, Won Joo Jeong, Sangkil Lee, Jin-Heon Jeong, Min-Ju Yeo, Jeong-Ho Hong, Hong-Kyun Park, Inyoung Chung, Beom Joon Kim, Jae Seung Bang, Hee-Joon Bae, Moon-Ku Han

**Affiliations:** 1 Department of Neurology, Seoul National University Bundang Hospital, Seoul National University College of Medicine, Seongnam, Korea; 2 Department of Neurology, Gyeongsang National University Changwon Hospital, Changwon, Korea; 3 Department of Critical Care, Seoul National University Bundang Hospital, Seongnam, Korea; 4 Department of Intensive Care Medicine and Neurology, Dong-A University Hospital, Busan, Korea; 5 Department of Neurology, Chungbuk National University Hospital, Chungju, Korea; 6 Department of Neurology, Keimyung University Dongsan Medical Center, Daegu, Korea; 7 Department of Neurosurgery, Seoul National University Bundang Hospital, Seongnam, Korea; Osaka University Graduate School of Medicine, JAPAN

## Abstract

**Background and objective:**

Symptomatic basilar artery stenosis (BAS) is associated with high risk of ischemic stroke recurrence. We aimed to investigate whether statin therapy might prevent the progression of symptomatic BAS and stroke recurrence.

**Methods:**

We retrospectively analyzed the data of patients with acute ischemia with symptomatic BAS, which was assessed using magnetic resonance angiogram (MRA) imaging on admission day, and 1 year later (or the day of the clinical event). The clinical endpoints were recurrent ischemic stroke and its composites, transient ischemic attack, coronary disease, and vascular death.

**Results:**

Of the 153 patients with symptomatic BAS, 114 (74.5%) were treated with a statin after experiencing a stroke. Statin therapy significantly prevented the progression of symptomatic BAS (7.0% vs 28.2%) and induced regression (22.8% vs 15.4%) compared to non-statin users (p = 0.002). There were 31 ischemic stroke incidences and 38 composite vascular events. Statin users showed significantly lower stroke recurrence (14.9% vs 35.9%, p = 0.05) and composite vascular events (17.5% vs 46.2%; odds ratio [OR], 0.29; 95% confidence interval [CI], 0.13–0.64) than those not using statins did. Recurrent stroke in the basilar territory and composite vascular events were more common in patients with progression of BAS than they were in other patients (OR, 5.16; 95% CI, 1.63–16.25 vs OR, 4.2; 95% CI, 1.56–11.34).

**Conclusion:**

Our study suggests that statin therapy may prevent the progression of symptomatic BAS and decrease the risk of subsequent ischemic stroke. Large randomized trials are needed to confirm this result.

## Introduction

Intracranial arterial stenosis (ICAS), an important cause of ischemic stroke, accounts for one-third of strokes in Asians who show a higher prevalence than Caucasians do. [1, 2] According to several Asian studies, intracranial arterial disease (IAD) accounts for 33–50%, 47%, 48%, and 10–25% of all stroke cases in China, Thailand, Singapore, and Korea, respectively.[3] Approximately one-third of posterior circulation strokes are caused by large artery stenosis of the vertebral, basilar, and posterior cerebral arteries. [4–6] The prevalence of ischemic stroke in patients with symptomatic basilar artery stenosis (BAS) was reported to be 8.7% in Korea. [7] In the Warfarin and Aspirin for Symptomatic Intracranial Disease (WASID) study, BAS had the highest stroke rate in any vascular and stenotic artery territory (15.0 and 10.7 per 100 patient-years, respectively). [8] Symptomatic ICAS is also known to frequently progress, which is related to increased risk of vascular events or death. [9]

Statins, which are 3-hydroxy-3-methylglutaryl coenzyme A reductase inhibitors, have been shown protective effect on progression of atherosclerosis in the coronary, carotid, and intracranial artery atherosclerosis. [10–12] Guidelines for the prevention of stroke in patients with stroke from the American heart association of 2014 recommended that statin therapy reduced should be initiated for the secondary prevention of patients with ischemic stroke or transient ischemic attack presumed to be of atherosclerotic origin. [13] Several studies revealed preventive effect of statin after ischemic stroke with or without symptomatic intracranial atherosclerosis include middle cerebral artery or basilar artery. [14–16] However, recent study showed the difference of plaque characteristics between anterior circulation and posterior circulation. [17] Therefore, we performed this study to retrospectively investigate the preventive effect of statin on the progression of symptomatic BAS and stroke recurrence.

## Methods

### Study population

Patients with BAS-induced acute ischemic stroke were retrospectively selected from the stroke registry of Seoul National University Bundang Hospital (SNUBH) from April 1, 2003 to June 30, 2014. Using the electronic chart review method, the records of the selected patients were further screened to determine if they fulfilled the inclusion or exclusion criteria. The inclusion criteria were: (1) acute ischemic stroke identified using brain magnetic resonance imaging (MRI) within 7 days after stroke onset, (2) large artery atherosclerosis according to the Trial of Org 10172 in Acute Stroke Treatment (TOAST) criteria, (3) BAS-induced ischemic stroke, and (4) existence of follow-up magnetic resonance angiography (MRA) image of the intracranial artery after an index stroke.

The exclusion criteria were: (1) embolic stroke or existence of high and medium risk of cardioembolism by trial of org 10172 in acute stroke treatment (TOAST classification) to investigate effect of statin on intracranial atherosclerosis. [18] (2) patients with vertebral artery stenosis; (3) nonatherosclerotic vasculopathy, such as dissection, vasculitis, and dissecting aneurysm; and (4) patients who underwent angioplasty or intravascular stenting. We collected the following information from recorded patient data: (1) demographics (age and sex); (2) medical history of transient ischemic attack or stroke; and (3) presence of cardiovascular risk factors such as hypertension (preadmission history and medical records), diabetes mellitus, dyslipidemia, and current cigarette smoking. The study was approved by the Seoul National University Bundang Hospital institutional review board.

### Baseline and clinical assessment

The National Institutes of Health Stroke Scale (NIHSS) was used by the neurologists to assess the patients on presentation and was rechecked by an experienced stroke physician assistant immediately after admission. Clinical and laboratory information including initial use of statins (HMG-CoA reductase inhibitors) after index stroke, systolic and diastolic blood pressure at admission, lipid profile, blood glucose and HbA1c at presentation, pre-stroke modified Rankin scales, and time interval from stroke onset to presentation, were collected and prospectively entered into the stroke registry database. Statin use was defined as positive if the patients received HMG-CoA reductase inhibitors for more than half of the time interval between the initial and the follow up MRA from the admission day after an index stroke.

Initial and follow-up MRA imaging was performed at our institution using same MRI protocol and 1.5-Tesla MRI scanners (Intera Achieva, Philips Medical Systems, Best, The Netherlands), consisting of three-dimensional (3-D) time-of-flight (TOF) sequences sensitive to arterial flow. On initial assessment, the diagnosis of symptomatic stenosis was made by experienced stroke neurologists and was confirmed by neuroradiologists. To assess the degree of BAS, the vessel was measured at its point of maximal narrowing, and the value was compared with that of a normal section of the basilar artery proximally adjacent to the stenosis using MRA (normal lumen diameter-residual lumen/normal lumen diameter). The BAS was graded as follows: mild (< 50%), moderate (> 50%), and severe (focal signal loss with the presence of a signal of distal basilar or posterior cerebral artery) stenosis, and occlusion. [16] We categorized the degree of stenosis as mild and severe (patients with < 50% and those with moderate stenosis to occlusion, respectively).

### Outcome measures

**Fig 1:**
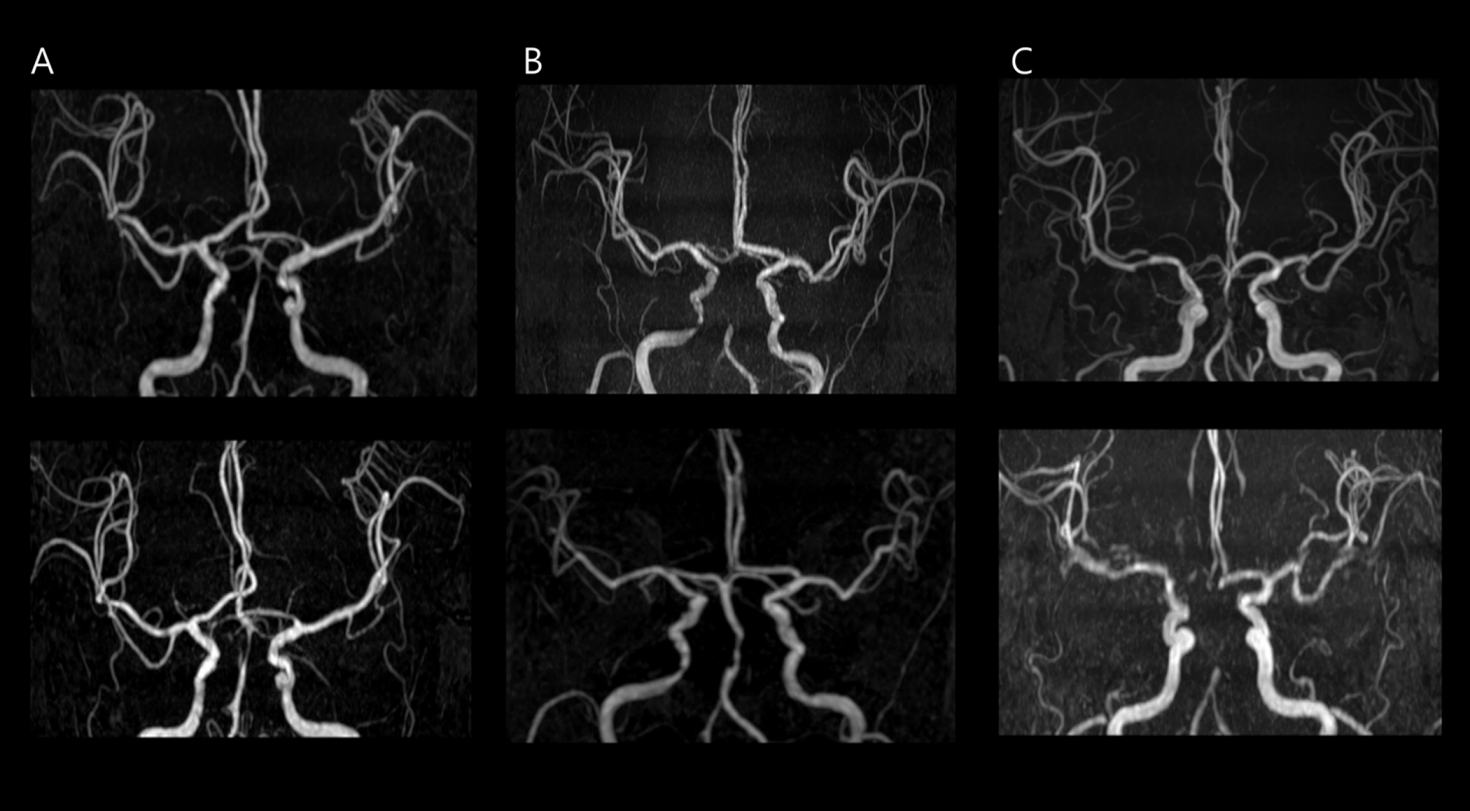
Baseline and follow-up three-dimensional (3D) time-of-flight magnetic resonance angiogram (MRA) of intracranial stenosis. A) Stationary, (B) regression, and (C) progression.

Follow-up MRA evaluations were performed within 12–24 months after an index stroke or the day of a recurrent stroke. As shown in Fig 1, progression was defined as worsening of stenosis by 1 or more grades on the final MRA compared with the baseline MRA, whereas regression was defined as an improvement of stenosis by 1 or more grades. The functional status and new symptoms of each patient were evaluated by the neurologists at an outpatient clinic or by a trained nurse using a structured telephone interview for 12 months after the stroke. The primary outcome was the progression of BAS a determined using an MRA. The occurrence of a recurrent stroke after an index stroke was the secondary outcome. Within 7 days after an index stroke, the systemic causes of clinical deterioration (e.g., infection) or worsening of initial symptoms (e.g., progression or hemorrhagic transformation) were not classified as a clinical recurrence but early neurologic deterioration.

A composite vascular event was defined as the occurrence of recurrent ischemic stroke, transient ischemic attack (TIA), acute coronary syndrome (ACS), or vascular death after an index stroke. TIA was defined based on clinical findings consistent with the occurrence of a stroke that lasted < 24 h without imaging evidence of acute infarction. ACS included any symptoms attributable to the obstruction of the coronary arteries (e.g., myocardial infarction and unstable angina). Vascular deaths included sudden death, death within 30 days of a cardiac event, or any sudden death that was not clearly due to nonvascular causes. The annual rate of clinical events including recurrent stroke and composite vascular events was calculated.

### Statistical analyses

All statistical analyses were performed using the statistical package for the social sciences (SPSS) for Windows software (version 21.0, SPSS Inc.). The univariate analysis was performed using Pearson chi-square test, Fisher’s exact method, Student’s *t*-test, or the Mann–Whitney U test as appropriate. Continuous variables are expressed as the mean ± standard deviation (SD) or the median (interquartile range, IQR), whereas categorical variables are presented as absolute values and percentages. The outcomes were compared using an ***X***^***2***^ test for categorical variables. All reported *p*-values are two-sided and were considered statistically significant at < 0.05.

## Results

Of the 447 consecutive patients who had an acute ischemic stroke within the territory of the basilar artery, 39 (8.7%) who underwent mechanical embolectomy, stenting, or angioplasty were excluded. Among the 408 patients who underwent TOF-MRA during an index stroke, 218 of them (44.8%) had BAS while 65 patients (14.5%) who did not undergo a follow-up MRA study were excluded. The 153 patients who fulfilled the inclusion criteria and were considered eligible consisted of 114 and 39 in the statin user (after index stroke) and non-statin user groups, respectively. In 39 non-statin users, 3 patients were not given statin less than half of the period of follow up by decision of physician. 26 patients did not administer statin due to low LDL cholesterol level. As shown in Table 1, no significant differences were noted in terms of age, sex, vascular risk factors, laboratory data, and vital signs except for the previous administration of statin, as well as triglyceride, and high-density lipoprotein cholesterol (HDL) levels, between the two groups. The initial severity of the stroke and vascular stenosis, as well as the initial choice of antithrombotic drug, did not differ significantly between both groups. The mean time interval between the initial and follow-up MRA was 14.6 ± 5.8 months.

**Table 1:** Baseline characteristics of patients who were treated with statin and those who were not.

	Total (n = 153)	Statin (n = 114)	Non-statin (n = 39)	p-value
**Age**	67.97±10.219	68.02±9.858	67.85±11.343	0.928
**Male–no. (%)**	83 (54.2%)	62 (54.4%)	21 (53.8%)	0.953
**Risk factors**				
Hypertension	121(79.1%)	91 (79.8%)	30 (76.9%)	0.701
Diabetes Mellitus	69 (45.1%)	52 (45.6%)	17 (43.6%)	0.826
Dyslipidemia	36 (23.5%)	27 (23.7%)	9 (23.1%)	0.938
Smoking	49 (32.0%)	38 (33.3%)	11 (28.2%)	0.554
Stroke	34 (22.2%)	24 (21.1%)	10 (25.6%)	0.552
**Prior statin therapy**	20 (13.1%)	19 (16.7%)	1 (2.6%)	0.024
**Glycosylated hemoglobin—%**	6.98±1.701	7.01±1.735	6.85±1.600	0.618
**TC(mg/dL)**^**†**^	187.15±41.876	186.14±43.430	190.10±37.330	0.612
**TG(mg/dL)**^**†**^	145.97±113.888	159.37±127.581	106.82±36.984	<0.001
**HDL-C(mg/dL)**^**†**^	43.61±11.636	42.30±11.343	47.46±11.774	0.016
**LDL-C(mg/dL)**^**†**^	111.36±32.381	113.44±33.799	105.28±27.317	0.175
**Blood pressure (mmHg)**				
Systolic	162.18±27.124	161.56±24.762	164.00±33.390	0.677
Diastolic	86.86±16.217	86.99±16.465	86.49±15.674	0.868
**NIHSS at admission**	4.11±2.968	3.90±2.765	4.72±3.464	0.140
**Stenosis of basilar artery**^††^				0.171
< 50%	85 (55.6%)	67 (58.8%)	18 (46.2%)	
≥ 50%/occlusion	68(44.4%)	47(41.2%)	21(53.8%)	
**Antithrombotics after index stroke**				
Antiplatelet agent	148 (96.7%)	112 (98.2%)	36 (92.3%)	0.072
Anticoagulant	15 (9.8%)	9 (7.9%)	6 (15.4%)	0.175

Abbreviations: TC = total cholesterol, TG = triglyceride, HDL_C = High-density lipoprotein cholesterol, LDL-C = low-density lipoprotein

cholesterol. Continuous data are presented as mean ± SD unless stated otherwise.

^†^Initial TC, TG, HDL-C and LDL-C were performed at admission day.

^††^Stenosis was estimated on initial MRA

### Change of arterial stenosis

The changing in the BAS degree based on lipid profile is presented in Table 2, and 51 of the 153 patients (33.3%) showed a change. Among the patients who were administered a statin after an index stroke, 26 (22.8%) regressed and eight (7.0%) progressed in the degree of BAS (Fig 1). The rate of progression of the BAS degree was significantly higher in the untreated group than it was in the group treated with statins (p = 0.002). The triglyceride level of the stationary group was significantly higher than that of the group that regressed and progressed group (p = 0.047), but the total cholesterol, triglyceride, HDL, and low-density lipoprotein cholesterol (LDL) levels of all the patients at follow-up were not different. The lipid profiles also did not differ between initial and follow-up values between the two groups.

**Table 2:** The change of basilar stenosis by lipid profile and clinical events.

	Total	Regression	Stationary	Progression	p-value
**Statin therapy after index stroke**					0.002
Yes	114	26 (22.8%)	80 (70.2%)	8 (7.0%)	
No	39	6 (15.4%)	22 (56.4%)	11 (28.2%)	
**Lipid profile of statin-treated patients**^**†**^					
TC(mg/dL)	144.43±33.810	132.96±34.789	148.18±33.308	145.13±30.903	0.138
Change of TC(mg/dL)*	-42.73±44.927	-50.85±48.634	-39.18±42.512	-51.00±56.715	0.434
TG(mg/dL)	127.6±64.882	101.35±47.573	137.46±68.888	116.75±51.889	0.047
Change of TG(mg/dL)*	-33.25±120.787	-36.04±69.747	-33.97±137.473	-17.13±75.871	0.256
HDL-C(mg/dL)	45.58±10.68	45.42±11.549	45.15±9.871	50.25±15.285	0.616
Change of HDL-C(mg/dL)*	3.29±10.044	2.23±10.786	3.36±10.166	6.00±5.904	0.641
LDL-C(mg/dL)	72.78±26.041	65.62±23.197	75.86±27.102	66.00±20.071	0.166
Change of LDL-C(mg/dL)*	-41.46±38.122	-49.27±45.084	-36.86±34.966	-60.88±38.005	0.116

Abbreviations: TC = total cholesterol, TG = triglyceride, HDL_C = High-density lipoprotein cholesterol, LDL-C = low-density lipoprotein cholesterol

^†^Studies were performed at day of recurrent stroke or 1 year after index stroke.

*Difference between initial and follow-up lipid profile

### Clinical outcome

During the follow-up period, there were 31 (20.3%) ischemic strokes and 38 (24.8%) composite cardiovascular outcomes (Table 3). Furthermore, 18 (11.8%) of ischemic strokes were recurrent in the basilar territory. The annual rate of ischemic stroke was 16.6%, recurrent stroke in the basilar territory was 9.1%, and the composite event was 20.4%. In patients with moderate or severe BAS on the initial TOF MRA (> 50% stenosis or occlusion) we found that recurrent stroke in any territory (32.8% vs 10.5%, p = 0.001), recurrence in the same territory (22.4% vs 3.5%, p < 0.001), and composite vascular events (34.3% vs 17.4%, p = 0.016) were significantly more common than they were in patients with mild BAS (< 50% stenosis). In patients on statin therapy after an index stroke, recurrent stroke in any territory (14.9% vs 35.9%, p = 0.05), recurrent stroke in the basilar artery territory (7.9% vs 23.1%, p = 0.011), and composite vascular events (17.5% vs 46.2%, p < 0.001) were significantly lower than they were in non-statin users. Early neurological deterioration within 7 days was not different between the groups. Recurrent stroke in the basilar territory (31.6% vs 8.2%, p = 0.004) and composite vascular events (52.63% vs 20.9%, p = 0.003) were more common in progressed group compared with non-progressed group significantly. Recurrent stroke in any territory (36.8% vs 17.9%, p-value = 0.055) were common in progressed patients, but the difference was not significant.

**Table 3:** Outcome of symptomatic basilar artery disease according to statin treatment.

	Degree of initial stenosis			Statin treatment			Change of stenosis		
	< 50% (n = 86)	≥50% to occlusion (n = 67)	OR (95% CI)	Statin (n = 114)	Non-statin (n = 39)	OR (95% CI)	Non-progress (n = 134)	Progress (n = 19)	OR (95% CI)
END7d	15 (17.4%)	14 (20.9%)	1.25 0.556–2.812)	20 (17.5%)	9 (23.1%)	0.709 (0.292–1.723)	25 (18.7%)	4 (21.1%)	1.163 (0.355–3.805)
Recurrent stroke in any territory	9 (10.5%)	22 (32.8%)	4.183 (1.773–9.868)*	17 (14.9%)	14 (35.9%)	0.313 (0.136–0.720)*	24 (17.9%)	7 (36.8%)	2.674 (0.953–7.500)
Recurrent stroke in basilar territory	3 (3.5%)	15 (22.4%)	7.981 (2.203–28.910)*	9 (7.9%)	9 (23.1%)	0.286 (0.104–0.784)*	11 (8.2%)	6 (31.6%)	5.161 (1.639–16.254)*
Composite vascular event^†^	15 (17.4%)	23 (34.3%)	2.474 (1.167–5.245)*	20 (17.5%)	18 (46.2%)	0.248 (0.1112–0.549)*	28 (20.9%)	10 (52.6%)	4.206 (1.560–11.345)*

Abbreviations: END7d = early neurologic deterioration within 7 days. OR = Odds ratio, CI = Confidence interval

^†^Composite vascular events included ischemic stroke, transient ischemic attack, acute coronary syndrome, and vascular death.

*A value of P was less than 0.05.

## Discussion

In this study, we investigated the effect of statin therapy on symptomatic BAS, with the aim of developing a preventive management strategy for the progression of BAS incidences that are thought to be related to clinical outcomes of symptomatic BAS. Our results indicate that statin therapy might prevent the progression of symptomatic BAS and reduce clinical events including ischemic strokes in any vascular territory, the same vascular territory, and composite vascular events after an index stroke.

In several previous observational studies, symptomatic intracranial artery atherosclerosis has shown dynamic change with progression and regression occurring in 9%–32.5% and 7.5%– 29%, respectively. [9, 19, 20] In our study, the patients on statin therapy showed lower progression rates and higher regression than the patients without statin treatment did. These findings indicate that statins had an effect on basilar artery atherosclerosis. The follow-up MRA after index strokes revealed no significant differences in the regression rate between the groups with and without statin therapy (22.8% and 15.4%, respectively). However, non-statin users showed a significantly higher rate of progression (28.2%) than the treated patients (7%) did. The patients with progression of BAS showed higher incidences of recurrent basilar strokes and composite vascular events than those without progression did, which indicates that statins prevented the progression of stenosis and the occurrence of clinical events in the symptomatic basilar disease.

The plaque characteristics are different between anterior circulation and posterior circulation. [17] Previous studies have found that statin therapy prevent recurrent stroke in the symptomatic intracranial atherosclerosis. [14, 16] These studies include patients with symptomatic atherosclerosis in the anterior circulation or posterior circulation. Symptomatic atherosclerosis in the posterior circulation, especially BA was included less than half in both studies. Therefore, our result indicates the preventive effect of statin therapy on symptomatic BAS.

In the SAMMPRIS study, the management of vascular risk factors includes achieving systolic blood pressure < 140 mmHg, LDL < 70 mg/dL, controlling other risk factors, and the institution of a lifestyle modification program that stabilizes the atherosclerotic plaque and prevents further progression of atherosclerosis. [14] In our study, both progressed and regressed patients showed low LDL< 70 mg/dL during the follow-up exam. The lipid profile result suggests that statins have more than a lipid-lowering effect and may also act on atherosclerotic stenosis.

The prevalence of clinical events in all the patients showed 31 (20.3%) incidences of recurrent stroke in any territory, 18 (11.8%) recurrent strokes in the basilar territory, and 38 (24.8%) composite vascular events. The annual rate of clinical events in our study was higher than previously reported for strokes with intracranial large artery disease. [8, 21, 22] In the study by Kim et al., [21] the annual event rate of symptomatic vertebral artery stenosis was 8.2% of all strokes in any territory, 4.1% of posterior circulation strokes, and 13.7% of composite cardiovascular outcomes.

In another study on BAS disease, the any-territory stroke recurrence, and same-territory stroke recurrence rates were 10.3% and 8.2% per year, respectively. [22] In a subgroup analysis of the WASID trial, the annual rates of clinical events were 15% and 10.7% for any and basilar territory strokes, respectively. [8] Although our study included patients with mild stenosis (< 50%), the annual rate of clinical events was higher than that reported in previous studies. [23, 24] The annual clinical events of the any-territory stroke, same-territory stroke, and composite cardiovascular outcomes in patients with severe stenosis (> 50%) in our subjects were 36.5%, 18.1%, and 38.9%, so the difference was higher. Several studies reported a predisposition for intracranial large artery disease in the Asian population. [23, 24] These results may be associated with differences in race and vascular territory. We reasoned that Asian patients with BAS show poor outcomes and, therefore, stain treatment may be an effective and important strategy.

Patients with severe stenotic basilar disease showed higher incidences of all the clinical events except for early neurologic deterioration within 7 days than other patients did. The degree of stenosis and time from the event is an important predictor of stroke recurrence. [25] Adequate medical therapy to regress the degree of stenosis is necessary, but our results did not show a significantly higher regression rate after statin treatment than before treatment. Noyes et al. reported that the regression of atherosclerotic plaque following statin therapy in studies documenting regression occurred after an average of 19.7 months. [26] Our mean observational period was less than the suggested period and, therefore, the regression rate of BAS may increase when the degree of stenosis is determined after a longer period than used in this study. Therefore, statin therapy might be a potential alternative management strategy for treating symptomatic BAS.

Our study has several limitations that are worth mentioning including the fact that it had a retrospective, small-sized, nonrandomized study design. Clinical and angiographic outcomes were obtained from follow up patients only. Therefore, the patients included in this study may constitute a high-risk group who experienced recurrent strokes after index strokes. Although treatment with anticoagulant and antiplatelet agents does not show a different outcome in large artery atherosclerosis, [27] the lack of standardization of the use of therapeutic agents including dose and duration of antithrombotics and statin within subjects is also a limitation. Due to the small size of our study, we were not able to perform multivariate analysis to comfirm association with outcome and statin therapy. Furthermore, the use of MRA imaging for the evaluation BAS is another limitation of this study. In the recent stroke outcomes and neuroimaging of intracranial atherosclerosis trial, the positive predictive MRA values were lower than those of computer tomographic angiography were. [28] Despite several limitations of our study described above, this study provides clinical effectiveness of statin on intracranial large artery atherosclerotic disease, especially in patients with symptomatic basilar artery stenosis.

The results of this retrospective observational study suggest that statins may prevent the progression of BAS and the recurrence of stroke and other cardiovascular events. Furthermore, it would be expedient to plan randomized clinical trials to confirm the exact effect of statins on symptomatic BAS and to establish an appropriate therapeutic regimen.

## Supporting information

S1 Fig Flow chart of enrolled subjects with symptomatic basilar disease. (TIF)

S1 File Minimum data set of our study population. (XLSX)
