# Supplementary figures and images for: Effect of statin on progression of symptomatic basilar artery stenosis and subsequent ischemic stroke
Source: PLoS One. 2017 Oct 11;12(10):e0183798. doi: 10.1371/journal.pone.0183798 (PMC5636063; doi:10.1371/journal.pone.0183798)

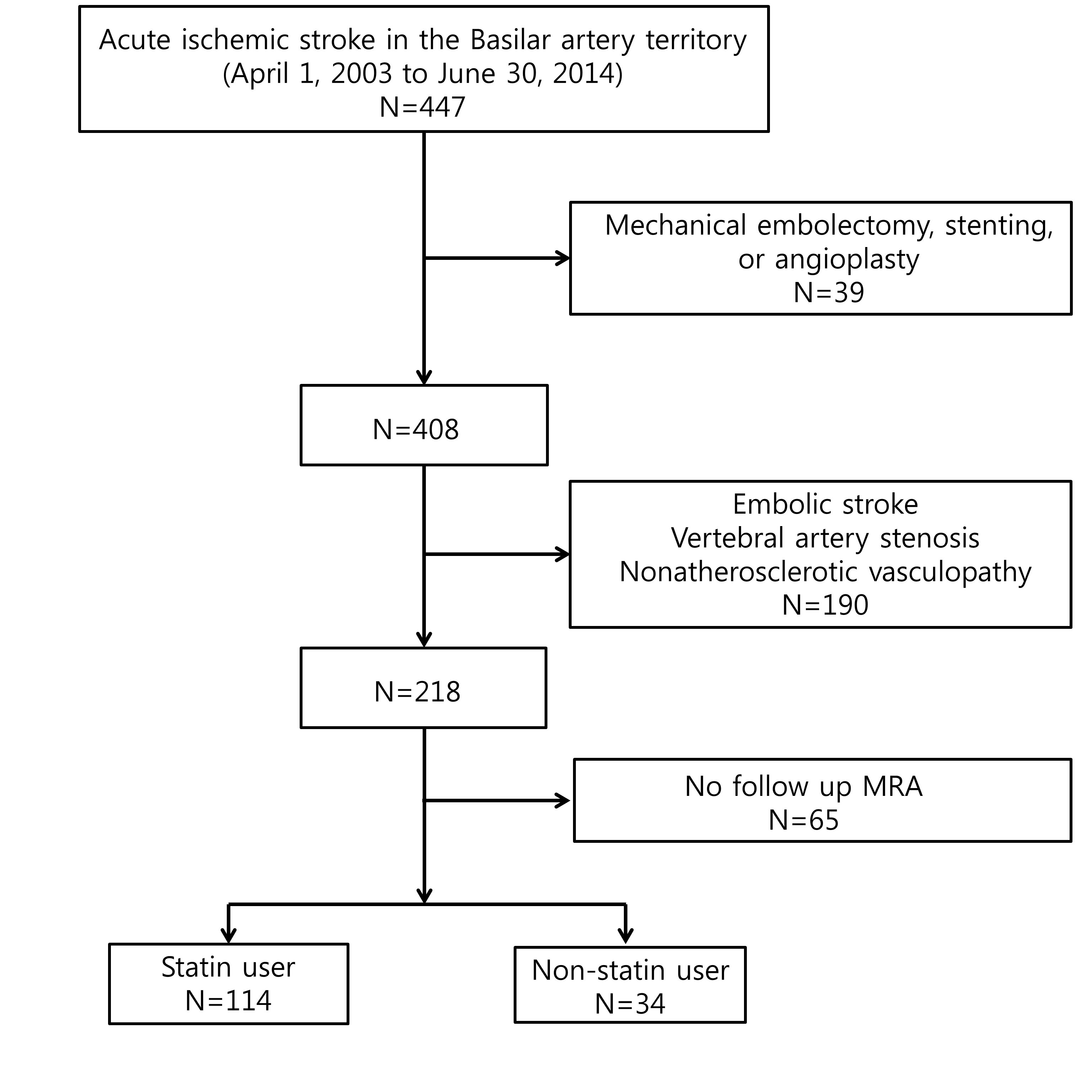

Supplement: S1 Fig — (TIF) [file pone.0183798.s001.tif]
